# Non-contact Quantification of Jugular Venous Pulse Waveforms from Skin Displacements

**DOI:** 10.1038/s41598-018-35483-4

**Published:** 2018-11-22

**Authors:** Emily J. Lam Po Tang, Amir HajiRassouliha, Martyn P. Nash, Poul M. F. Nielsen, Andrew J. Taberner, Yusuf O. Cakmak

**Affiliations:** 1 Auckland Bioengineering Institute, The University of Auckland, Auckland, New Zealand; 2 Department of Engineering Science, The University of Auckland, Auckland, New Zealand; 3 Department of Anatomy, University of Otago, Dunedin, New Zealand

## Abstract

The jugular venous (JV) pressure waveform is a non-invasive, proven indicator of cardiovascular disease. Conventional clinical methods for assessing these waveforms are often overlooked because they require specialised expertise, and are invasive and expensive to implement. Recently, image-based methods have been used to quantify JV pulsation waveforms on the skin as an indirect way of estimating the pressure waveforms. However, these existing image-based methods cannot explicitly measure skin deformations and rely on the use of photoplethysmography (PPG) devices for identification of the pulsatile waveforms. As a result, they often have limited accuracy and robustness and are unsuitable in the clinical environment. Here, we propose a technique to directly measure skin deformations caused by the JV pulse using a very accurate subpixel registration algorithm. The method simply requires images obtained from the subject’s neck using a commodity camera. The results show that our measured waveforms contained all of the essential features of diagnostic JV waveforms in all of 19 healthy subjects tested in this study, indicating a significantly important capability for a potential future diagnostic device. The shape of our measured JV displacement waveforms was validated using waveforms measured with a laser displacement sensor, where the average correlation score between the two waveforms was 0.93 ± 0.05. In addition, synchronously recorded ECG signals were used to verify the timings of diagnostic features of the measured waveforms. To our knowledge, this is the first use of image registration for direct measurement of JV displacement waveforms. Significant advantages of our novel method include the high precision of our measurements, and the ability to use ordinary cameras, such as those in modern mobile phones. These advantages will enable the development of affordable and accessible devices to measure JV waveforms for cardiac diagnostics in the clinical environment. Future devices based on this technology may provide viable options for telemedicine applications, point of care diagnostics, and mobile-based cardiac health monitoring systems.

## Introduction

Cardiovascular disease is a leading cause of death worldwide, and was responsible for 31 % of all deaths in 2015^1^. There is a need to develop methods and devices that can quickly and non-invasively alert clinicians to disorders of the cardiovascular system.

Changes in fluid pressures within blood vessels of the circulatory system reflect the mechanical function of the heart. By examining the pressures developed within the major arteries or veins, clinicians can infer information about the heart’s activity. In particular, analysis of the jugular venous (JV) pressure waveform can indicate a variety of diseases associated with the right heart chambers, such as pulmonary hypertension, tricuspid regurgitation, and constrictive pericarditis^2^.

**Figure 1:**
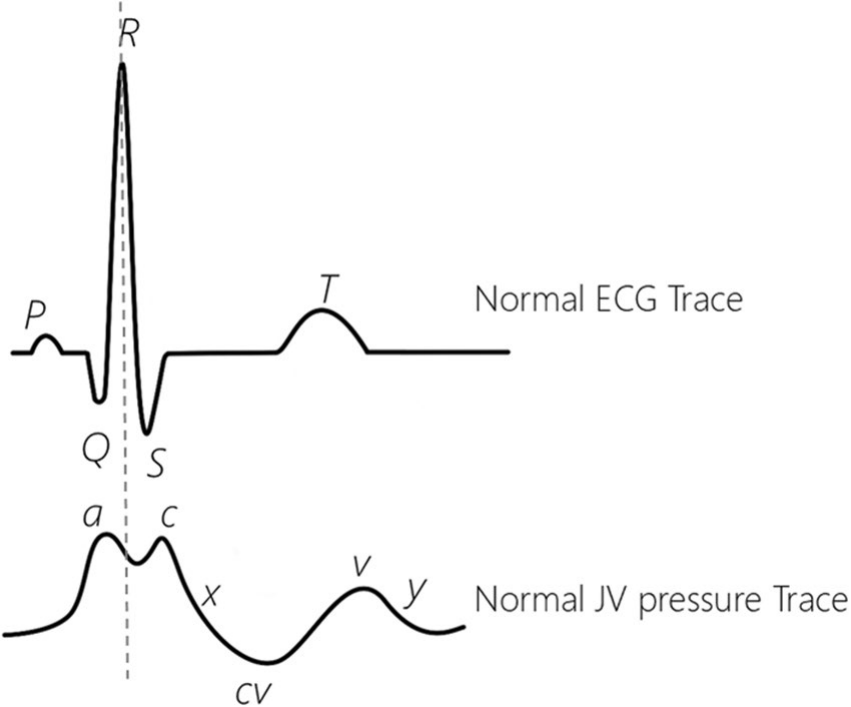
A normal ECG trace (on the top) and Jugular venous (JV) pressure waveforms (on the bottom). In a normal JV pressure trace the R peak of the ECG signal happens between the a peak and the c peak of the JV waveform. The waveforms are adapted from^3,4,8^.

The JV pressure waveforms of healthy subjects have three distinct pressure peaks, commonly labelled as the *a*, *c* and *v* peaks (Fig. 1), and two pressure descents (labelled *x* and *y*). The *a* peak represents atrial contraction, and the *x* descent represents atrial relaxation. The *c* peak represents the closure of the tricuspid valve and right ventricular contraction, and the *v* peak represents right atrial filling. At the beginning of diastole, the tricuspid valve opens causing blood to flow from the right atrium into the right ventricle. This is seen as a decrease in JV pressure represented by the *y* descent^3^.

Abnormalities of the JV pressure waveform are a sign of likely cardiac dysfunction. Physical changes in the structure of the right heart chambers, heart valves, and blood vessels can also lead to irregularities in this waveform^2^. For example, regurgitation of blood through a damaged tricuspid valve can be identified by merged *c* and *v* peaks during ventricular contraction^4,5^. The JV pressure waveform is thus an effective clinical indicator of the condition of the right side of the heart and vasculature.

The most direct way to observe the JV pressure waveform is via catheterisation, which involves inserting a pressure sensor into the vessel. This invasive procedure requires surgical expertise and is not performed routinely, but is typically reserved for patients who require medium term monitoring^5^. Changes in the fluid pressure within blood vessels causes deformations of the surrounding tissues and skin, particularly where vessels are close to the body surface. Visual observation of skin displacements in the proximity of the JV is a clinically established technique to evaluate the JV pressure waveforms^6^. Most commonly, dynamic deformation of the JV in the anterolateral side of the neck, which returns blood to the right atrium^7^, is used to assess the condition of the heart. However, because this method requires clinical expertise and is difficult to master, it is often neglected, or performed poorly^3,9^. During inspection with the naked eye, it is a challenge to distinguish the features of JV pressure waveforms as the *a*, *c* and *v* peaks appear rapidly in succession^3^. Due to difficulties in directly measuring pressure waveforms, or estimating their form from visual inspection of skin pulsation, alternative methods have been developed to measure the skin deformation waveforms caused by changes of pressure inside the JV or carotid artery (CA).

Ultrasound imaging is a non-invasive technique that has been used to monitor the function of arteries and veins. Ultrasound can be used to measure changes in the internal diameter of the JV or CA, from which changes of pressure in the vessel due to pulsatile blood flow can be inferred^10,11^. These methods require specialised, expensive equipment and an experienced ultrasound technologist to position the recording probe appropriately. Furthermore, with ultrasound imaging, the probe must be in contact with the patient, which potentially distorts the signal due to the applied pressure and introduces a source of inter-operator variation in signal acquisition.

Recently, non-contact approaches have been proposed to measure the deformation of the skin overlying the JV and the CA as an indication of the pressure. These methods assume that the deformation waveform shares similar features to the pressure waveform. Amelard *et al*.^5^, Moco *et al*.^12^, and HajiRassouliha *et al*.^13^ have used non-contact techniques to measure JV or CA deformation waveforms. The technique of Amelard *et al*.^5^ is based on using photoplethysmography (PPG) imaging and a colour camera to extract the JV waveforms^5^. PPG imaging is an optical technique to measure changes in blood volume based on changes in the transmitted or reflected light from the skin. In typical PPG systems, a light emitting diode (LED) is used to illuminate the skin and the intensity of reflected or transmitted light is measured using a photodetector. Moco *et al*.^12^ proposed a method to measure cardiac-related frequency components of skin motion under nonuniform light using a colour camera, assuming that the motion is dominated by CA wall displacements. An alternative approach was proposed by HajiRassouliha *et al*.^13^, who used a monochrome camera to directly measure skin deformations using subpixel image registration.

PPG imaging used by Amelard *et al*.^5^ involves measuring the intensity of visible or infrared light reflected from the patient’s neck to estimate skin deformation waveforms caused by the JV. This technique typically requires a finger PPG device for identification of the pulsatile waveforms related to the jugular vein (in addition to the neck PPG imaging device). The PPG imaging study of Amelard *et al*.^5^, reported that the *a* peak of the JV waveform was visible in only half of the subjects, which indicates the lack of sensitivity of these methods. Moreover, the proposed method of Amelard *et al*.^5^ required a stand to hold the camera (i.e. the imaging device) at a distance of 1.5 m above the patient, which may be inconvenient in some clinical situations. The method of Moco *et al*.^11^ required non-uniform illumination of the neck to increase variations in the frame-to-frame contrast of the moving regions. This method involves some manual steps, and has not yet been validated against an independent reference method.

The subpixel image registration method of HajiRassouliha *et al*.^13^ has some advantages compared to the PPG imaging methods of Amelard *et al*.^5^ and the skin-motion imaging of Moco *et al*.^12^. While PPG imaging^5^ and skin-motion imaging^12^ infer skin movement from changes in pixel colour intensity in video recordings of the skin, the method of HajiRassouliha *et al*.^13^ uses a subpixel image registration algorithm to directly measure skin deformations caused by the CA pulsation on the neck using pixel intensity values. Moreover, unlike the PPG imaging methods of Amelard *et al*.^5^, the method of HajiRassouliha *et al*.^13^ does not rely on finger PPG recordings for identification of the pulse.

To address the limitations of PPG-based imaging, we have developed a novel non-contact technique that uses subpixel image registration to quantify skin displacement waveforms due to JV pulsation. We build on the approach proposed in HajiRassouliha *et al*.^13^ for quantifying CA waveforms. Our subpixel image registration algorithm (phase-based Savitzky-Golay gradient correlation (P-SG-GC)^14^) is able to measure skin deformations with unprecedented precision^15^. The P-SG-GC algorithm is a two-step procedure that determines the integer and subpixel components of displacement separately. In the first step, the integer shift is found using a novel gradient correlation method based on Savitzky-Golay differentiators. In the second step the integer shift is compensated for and the subpixel shift is found using the phase of the signal in the frequency domain. Here, we use this algorithm to measure skin deformations that arise from changes in JV pressure. Our device uses an off-the-shelf camera, and is non-contact, inexpensive, transportable and easy-to-use. We are not aware of any previous study that has used an image registration technique to measure skin deformations arising from the JV pressure waveforms.

## Methods

### Experimental methods

**Figure 2:**
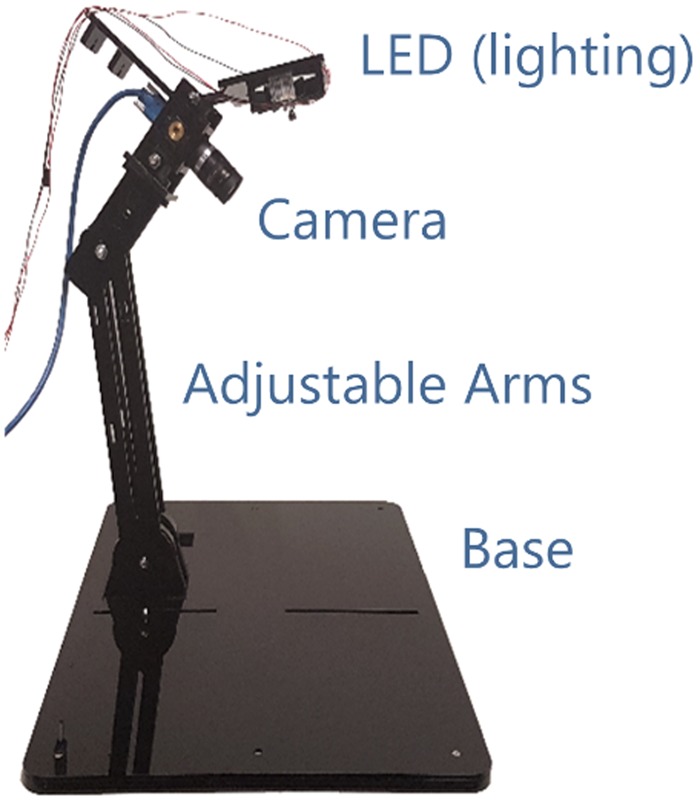
The camera rig used to record the videos from the JV pulsation of the subjects. The two movable arms helps to adjust the camera position relative to the neck. A blue LED was used to illuminate the location of the recordings.

This study was approved by the Otago University human ethics committee (reference number D17/127, category B, 4/4/2017). All experiments were performed in accordance with the guidelines and regulations of the Otago University human ethics committee. Our device consists of a custom-built rig that holds a camera (FLIR USB3 Flea3, FL3-U3-13Y3M-C) at approximately 30 cm from the subject’s neck and appropriate location with respect to the jugular vein, and software to measure the skin deformations and extract the JV displacement waveforms from the video image sequences (Fig. 2). This device has two movable arms in order to adjust the camera position relative to the neck. This allows use of the device for different neck sizes, different vessel positions, and for different areas of interest. A blue LED was used to enhance the contrast of intrinsic features of the skin, as melanin absorbs short wavelengths of the visible light (such as blue light) to a greater extent than it does for long wavelengths (such as red light)^16^. Even though the choice of a blue LED improves the robustness of the algorithm, the accuracy of the measurements is not dependent on the choice of LED colour. The position of the LED can also be adjusted to control contrast.

**Figure 3:**
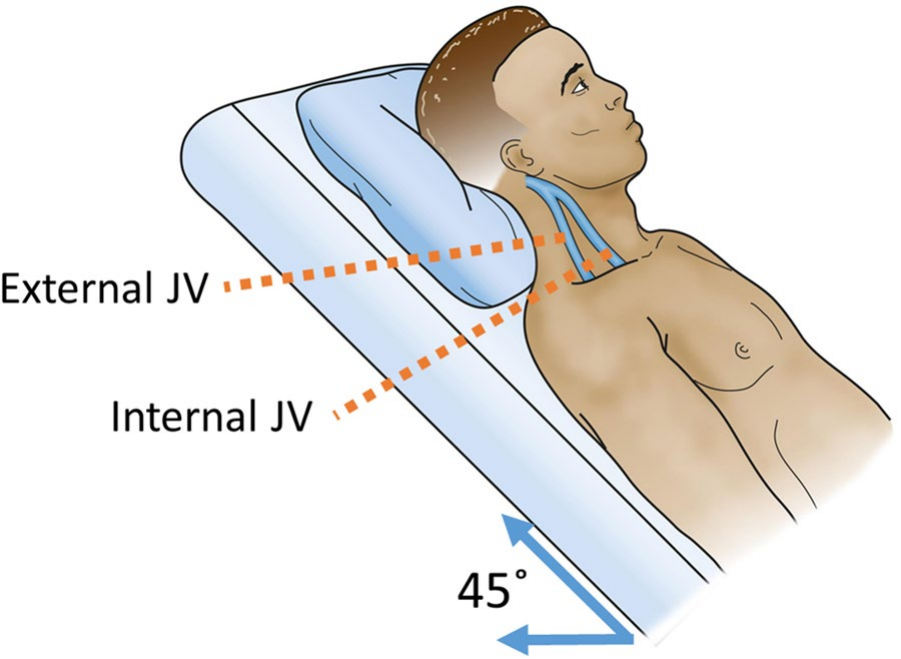
The typical position of the patient is 45° when evaluating jugular venous (JV) pressure in clinical examinations. This angle simplifies the process of estimating the blood pressure waveforms in the traditional method of using visual observations.

**Figure 4:**
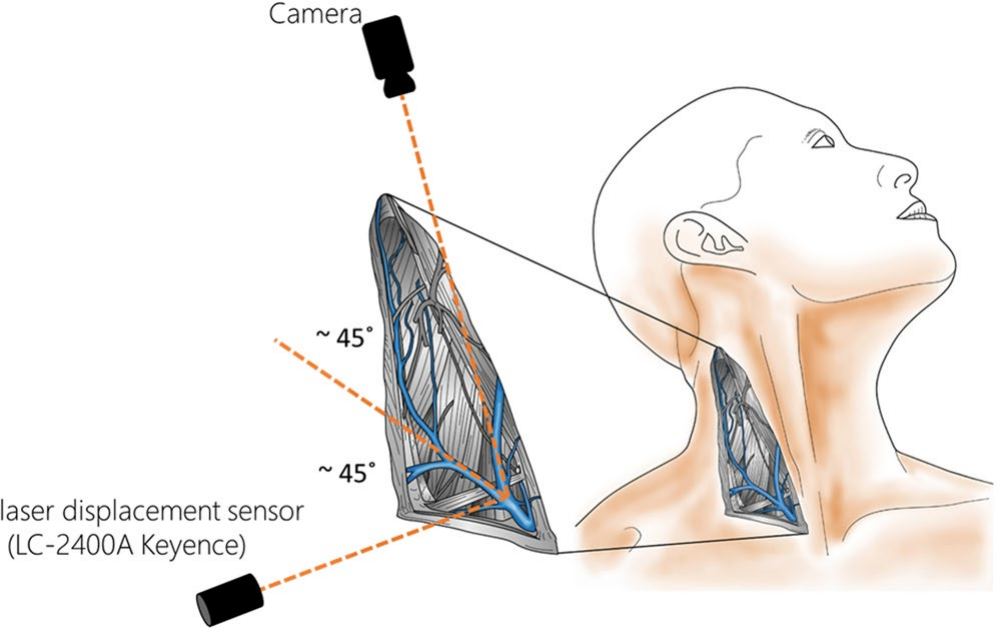
The experimental set-up used to record videos from the JV pulsation area. A laser displacement sensor was used to validate the displacement measurements of our method. The camera and the laser displacement measurement sensor were placed at approximately 45° with respect to the skin surface, and perpendicular to each other to avoid perspective distortion effects in the camera view.

Data were collected from 19 healthy participants (7 females), who had given informed consent. The participants were aged from 21 years to 63 years (mean age 25.5 years). The body mass index of the subjects ranged from 16.2 kg/m^2^ to 25.6 kg/m^2^ (mean 22.5 kg/m^2^). Demographic data for each subject are shown in Table 1. Conventional clinical examination of the vessels is typically conducted with a 45° seated angle between the torso and legs of the subject^3^ (Fig. 3). However, the JV pulse is rarely visible on the neck of a healthy subject in this position^17^. On the other hand, when a subject is placed in the supine position, the pressure in the venous system at the neck is greater and allows the pulse to be more visible on the surface of the skin^5^. In our experiments, subjects were thus placed in the supine position during imaging. The angle between the camera and the skin surface on the subject’s neck was manually set at approximately 45° (Fig. 4). Video recordings of the neck were captured at 90 frames per second with a resolution of 1280 pixel by 1024 pixel for a duration of 5 seconds.

**Table 1:** Descriptive data for the subjects used in this study.

Subject	Gender	Age (years)	Body mass index
1	F	21	24.5
2	F	21	24.7
3	M	28	24.2
4	M	27	23.4
5	M	35	20.4
6	M	23	25.6
7	F	21	16.2
8	M	23	20.8
9	M	22	24.4
10	M	22	21.4
11	F	23	19.5
12	M	21	24.1
13	M	23	20.1
14	F	22	23.2
15	F	22	24.9
16	M	24	23.7
17	M	63	22.5
18	F	22	21.1
19	M	21	22.9

A laser displacement sensor (LC-2400A laser displacement meter, and LC-2440 laser displacement head, Keyence), with a resolution of 0.2 µm, was used to provide a reference recording with which to compare the skin displacement measurements made using our camera device. The laser sensor was arranged at 90° to the camera, approximately 20 mm from the skin, and was directed at a location near the centre of the area of maximum pulsation on the top of the left clavicle of the subjects (Fig. 4). The 90° angle between the camera and the laser sensor was chosen to avoid perspective distortion effects in the camera view. Data from a 3-lead chest ECG were also recorded in 3 cases for timing validation. ECG and laser sensor recordings were acquired simultaneously using a National Instruments USB-6002 DAQ. Camera, ECG and laser recordings were captured simultaneously using a second USB-6002 DAQ with custom software written in LabVIEW 2016 (National Instruments). The ECG and laser sensor data were filtered using a first order low pass Butterworth filter with a cut-off frequency of 100 Hz.

### Data Analysis

**Figure 5:**
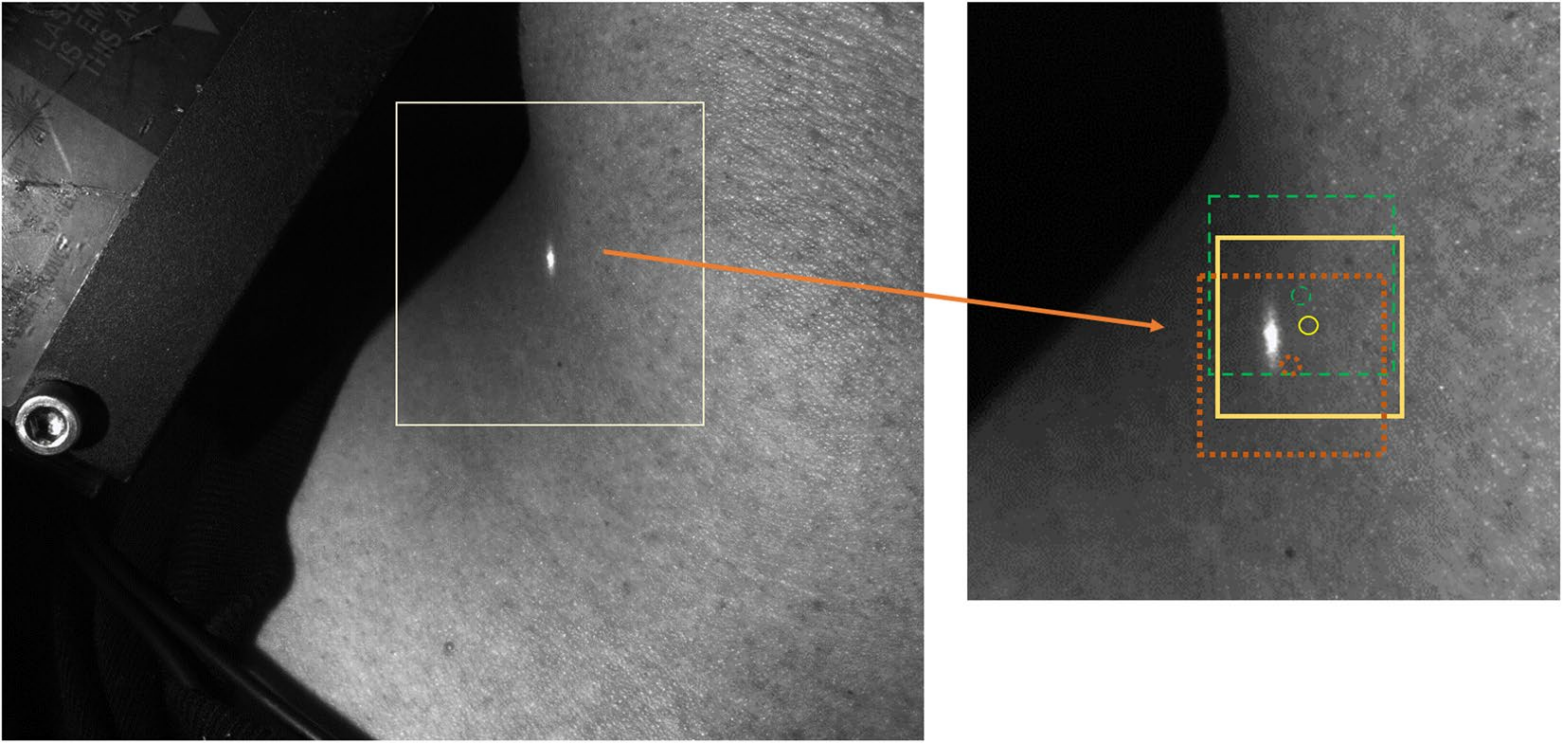
Example frame showing three subimages and their associated control points (circles). The neck covers approximately two-thirds of the image. The region of interested is cropped and zoomed on the right image for visualisation purposes. The bright spot visible in the images arises from the laser displacement sensor.

The deformation field across the image was found after dividing each image into smaller subimages. This process resulted in a single estimate of displacement between frames for each subimage, which was then attributed to a control point located at the centre of the subimage (Fig. 5). For this application, a 64 pixel by 64 pixel subimage size was used with an overlap of 49 pixel between subimages, resulting in 5780 control points across the entire field of view. The relatively small subimage size, and large relative overlap enables small localised deformations to be measured at a high density of points. Prior to further processing, a polygonal region of interest was manually drawn over the field of view, in order to exclude control points that were not located on the neck. The displacement of each remaining subimage was then estimated from frame to frame using the P-SG-GC algorithm^14^. The displacements were displayed as functions of time, to produce a displacement waveform for each control point.

**Figure 6:**
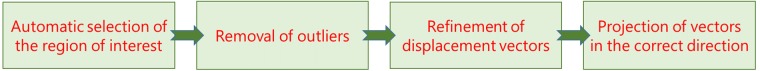
A system diagram showing the four main steps of our method for measuring the JV displacement waveforms.

To identify the skin displacement waveforms due to pulsatile venous blood flow, four main steps were taken. Figure 6 illustrates the workflow of our method for measuring the JV displacement waveforms, and the steps are described in the following sections.

### Automatic selection of the region of pulsation using frequency analysis

**Figure 7:**
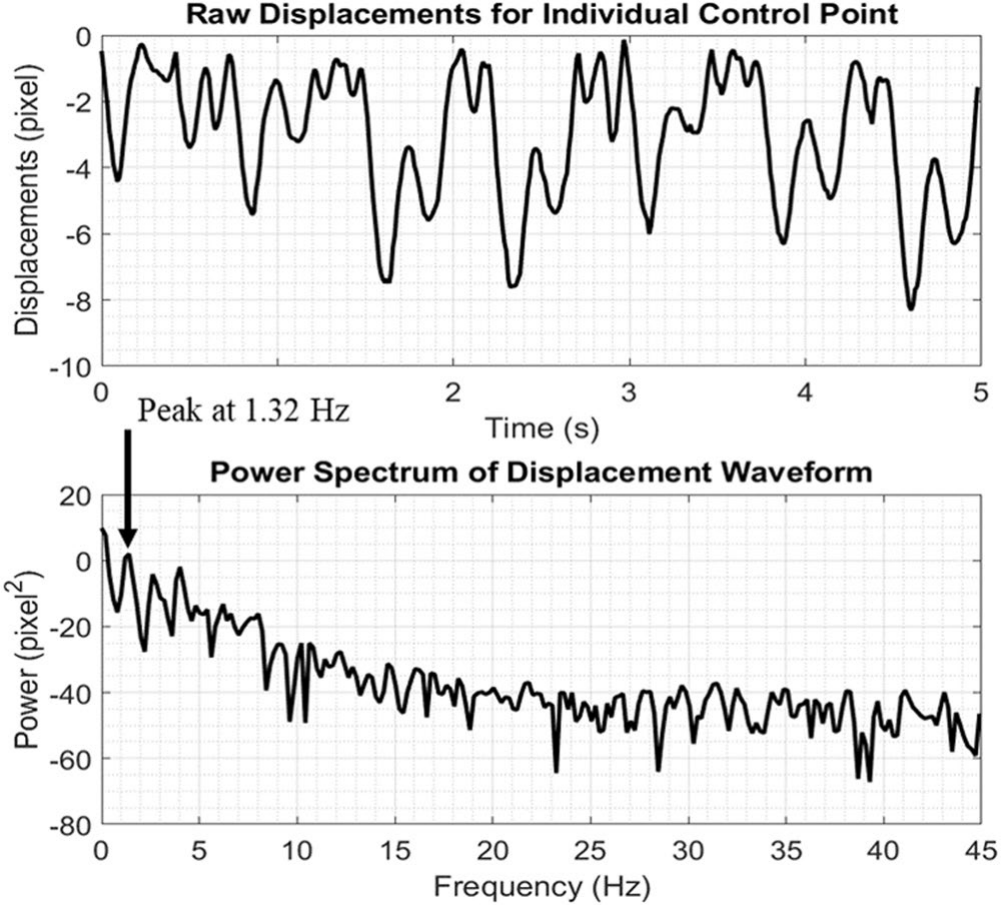
A displacement waveform and its fast Fourier transform (FFT). The maximum power for this waveform was at a frequency of 1.32 Hz. The top 5 % frequencies were used to filter the waveforms, which are more likely to be due to the JV pulsation.

Frequency analyses were conducted on waveforms measured for each of the control points. At each point, the LabVIEW *extract single tone information* module was used to identify the displacement frequency with the highest power within the frequency range of 0.7 Hz to 2 Hz (Fig. 7). This range is equivalent to a heart rate of between 42 beats per minute and 120 beats per minute, which encompasses the normal resting heart rate of adult humans, including athletes^18^. The waveforms were then ranked from maximum to minimum power, and the top 5 % were selected for further processing, as they were more likely to be related to subimages located over the JV. This percentage was chosen because, in our recordings, the skin overlying the JV covered approximately 5 % of the neck visible in our images.

### Removal of outliers using the signal derivative

The point-by-point time derivatives of the selected waveforms was used to further reduce the number of control points in order to find those that more reliably represented the pulsation of the JV. Large derivative values were not expected, since displacements do not change substantially over time from one image to the next. In our experiments, derivatives of JV waveforms that were distinctly periodic and noise-free were smaller than 0.25 pixel per frame. Therefore, we chose to discard waveforms with a derivative magnitude of greater than 0.25 pixel per frame.

### Refinement of displacement vectors based on the signal magnitude

**Figure 8:**
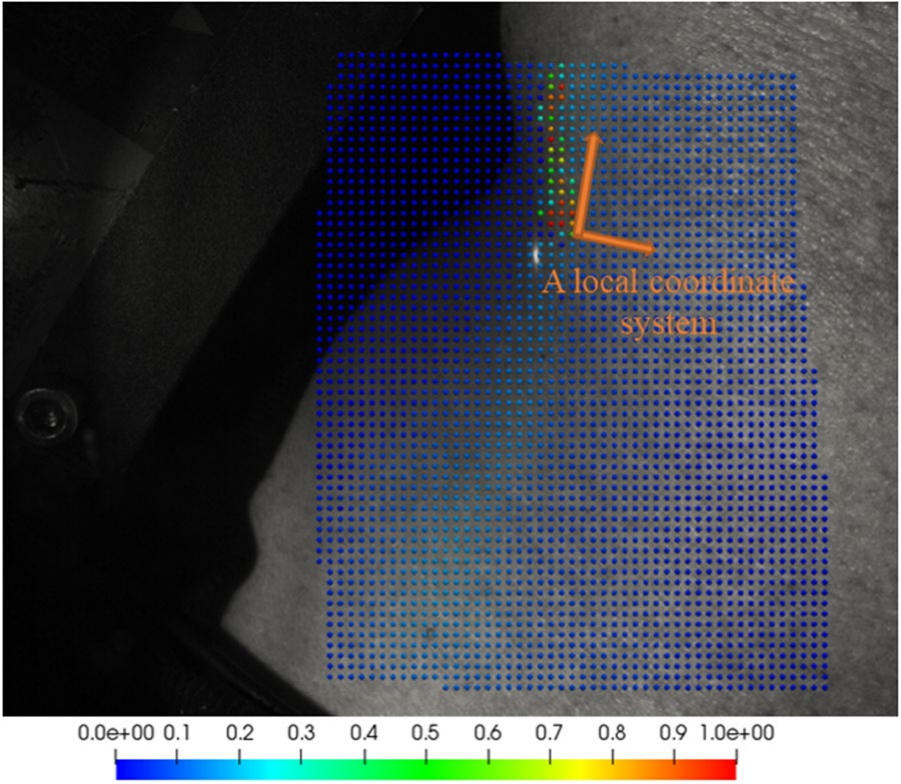
Power amplitude (normalised) at tone frequency (between 0.7 Hz and 2 Hz) of control points close to the jugular vein. The red and green dots that have larger amplitudes compared to the rest of the measurements shown the region of JV pulsation in this recording.

The control points located on or near the JV had the largest displacements, and typically arose from a strip of approximately 10 subimages in our images (Fig. 8). Thus, we selected the 10 waveforms with the largest amplitudes from the top 5 % of waveforms in step 1. If the selection consisted of fewer than 10 control points, then all points were used. The magnitudes of the displacement vectors for these control points were then averaged in the *x* and *y* directions to create an average JV waveform for each direction.

### Projection of vectors in the correct direction

The displacements of the control points are measured relative to their initial positions, and the direction of displacement vectors are defined in comparison to the first image. The coordinate system of the vectors is thus based the camera image, which is not fixed relative to the neck across subjects. Therefore, using the *x* or *y* components would be arbitrary, since the orientation of the subject with respect to the camera may be different in different recordings or from subject to subject. Using only the magnitudes of the vectors would not indicate all of the features of JV displacement waveforms. To address this, a local coordinate system, defined by the direction of pulsation was used to reorient the vectors.

To define the local coordinate system, from the remaining control points, the displacement vector with the maximum magnitude over the whole image and over time was identified (note that the outliers had been removed in previous steps). A sample local coordinate system is illustrated in Fig. 8. The vector with the maximum magnitude is associated with the subimage with the largest displacement, which is likely to be close to the area of pulsation. Therefore, this vector was used as an estimate of the direction of skin displacements at the area of pulsation, and the vector perpendicular to this was chosen to represent the orientation of the vessel. It was assumed that the area of interest was small enough such that no significant change in direction of the vessel within the region of interest would be observed. The remaining vectors were projected onto the vector with the maximum amplitude to make them normal to the vessel. The projected vectors were averaged, and were plotted over time to produce JV displacement waveforms.

### Validation

#### Validating the shape of the signal

To validate the shape of the displacement waveforms, they were compared to laser displacement data as described in^12^. Briefly, each measurement was normalised to it largest value over the entire 5 seconds recording, and the Pearson’s correlation coefficient was calculated as a correlation score using Equation 1^19^.

1 $${r}_{x,y}=\frac{N(\sum {x}_{n}{y}_{n})-(\sum {x}_{n})(\sum {y}_{n})}{\sqrt{[N\sum {{x}_{n}}^{2}-{(\sum {x}_{n})}^{2}][N\sum {{y}_{n}}^{2}-{(\sum {y}_{n})}^{2}]}}$$

where *r*_*xy*_ is the cross-correlation score, *x*_*n*_ and *y*_*n*_ are the signal values at frame *n*, and *N* is the total number of frames.

#### Validating the timing of the signal

The ECG information captured using a 3-lead chest ECG was used as another validation step to identify whether the characteristic peaks (*a*, *c*, and *v*) in the JV displacement waveforms occurred at the expected times during the cardiac cycle.

## Results

### Shape of the signal

**Figure 9:**
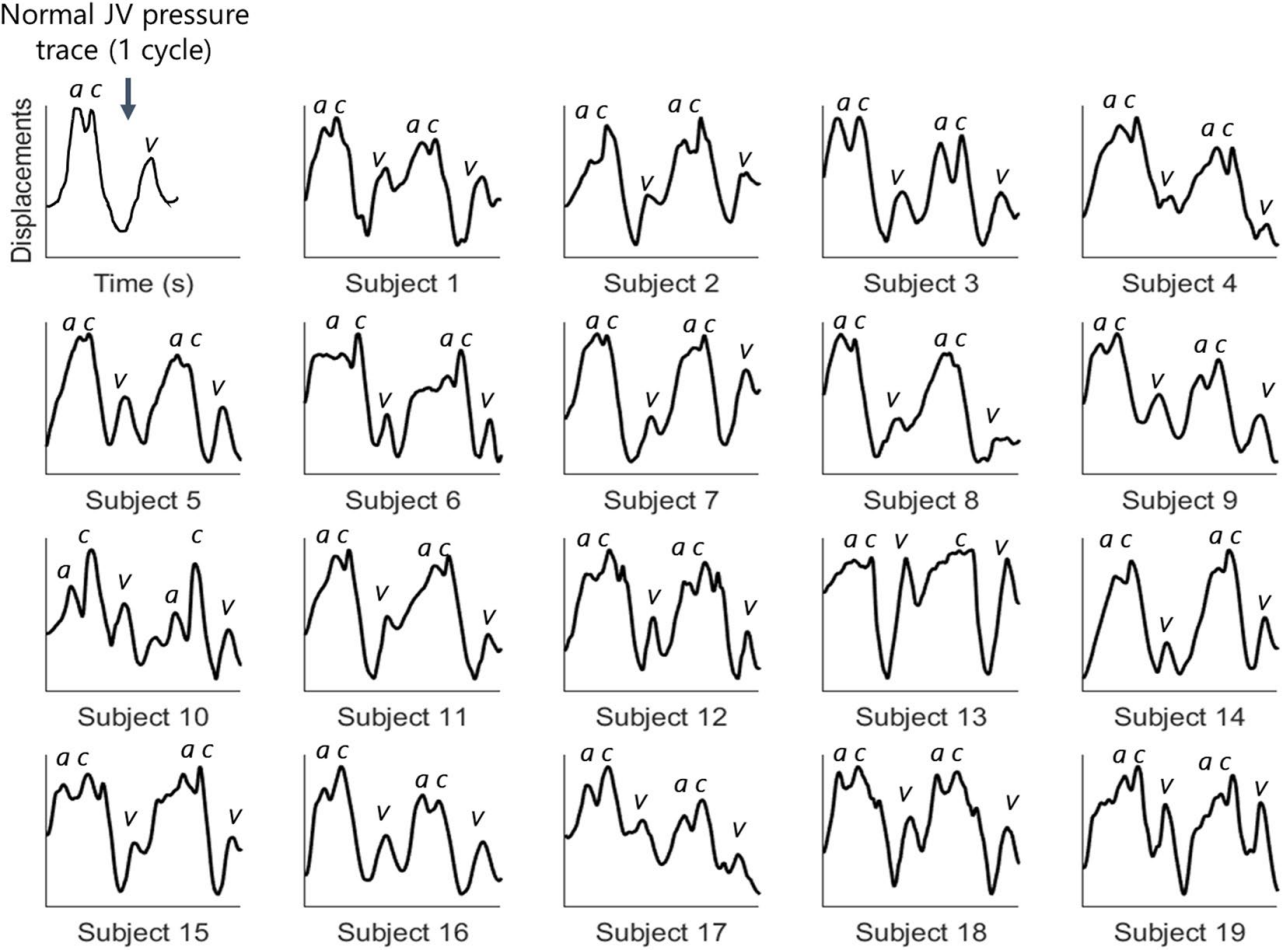
Jugular venous pulse displacement waveforms over two cardiac cycles for each of 19 healthy subjects. The three important peaks of the JV waveform (a, c, and v) were observed in all the subjects. The second cardiac cycle of Subject 13 was the only waveform that did not have one of the peaks (the a peak is missing).

**Figure 10:**
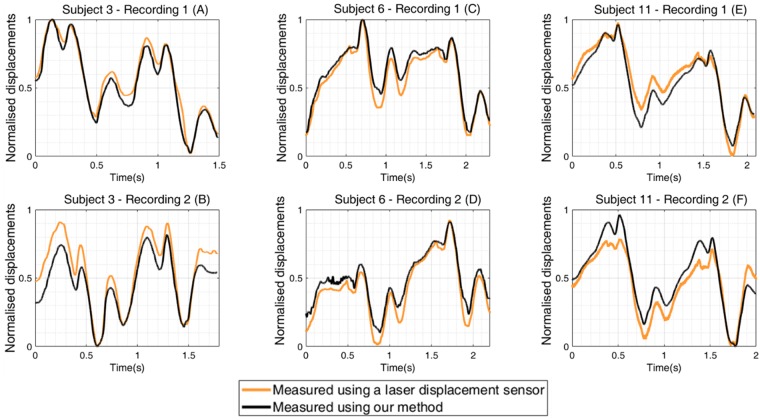
Normalised jugular vein displacement waveforms measured using our method (black lines) and using a laser displacement sensor (orange lines) derived from two recordings in each of three subjects. The correlation scores between the associated pairs of recordings in panels A to F were 0.98, 0.97, 0.96, 0.99, 0.96, and 0.95, respectively.

The JV displacement waveforms, measured for 19 healthy subjects each over two cardiac cycles, are illustrated in Fig. 9. The measurements indicate all of the characteristics of JV waveforms reported in recent literature. Healthy subjects typically exhibit three distinct peaks in the JV pressure waveforms, two of which are more prominent (*c* and *v*). The *c* and *v* peaks were identified in all subjects’ waveforms. The *a* peak is also present in the displacement waveforms, although it was not prominent in the second cycle of Subject 13, which contains an unusually large *v* peak relative to the *a* and *c* peaks. This could be due to some experimental errors, such as specular reflection, saturation of pixels, or movement of the subject. Otherwise, all of the *a*, *c*, and *v* peaks were present in all of the other displacement waveforms. The laser displacement data were matched well by the measured displacement waveforms using our method. The correlation score for 32 sets of data from 8 subjects was 0.93 ± 0.05, indicating that there is a strong correlation between the two methods for measuring JV displacement waveforms. Six examples of waveform measurements using our device and using the laser sensor are illustrated in Fig. 10. The correlation scores ranged from 0.95 in Fig. 10(F) to 0.99 in Fig. 10(D). These examples represent measurements performed using two videos recorded from each subject at different times. For each subject, the two waveforms contained consistent shape characteristics. A direct quantitative analysis of the similarity between the two waveforms is not conducted, since the measurements were not simultaneous, and the subjects’ heart beats and blood pressures were not constant.

## Timing of the signal

**Figure 11:**
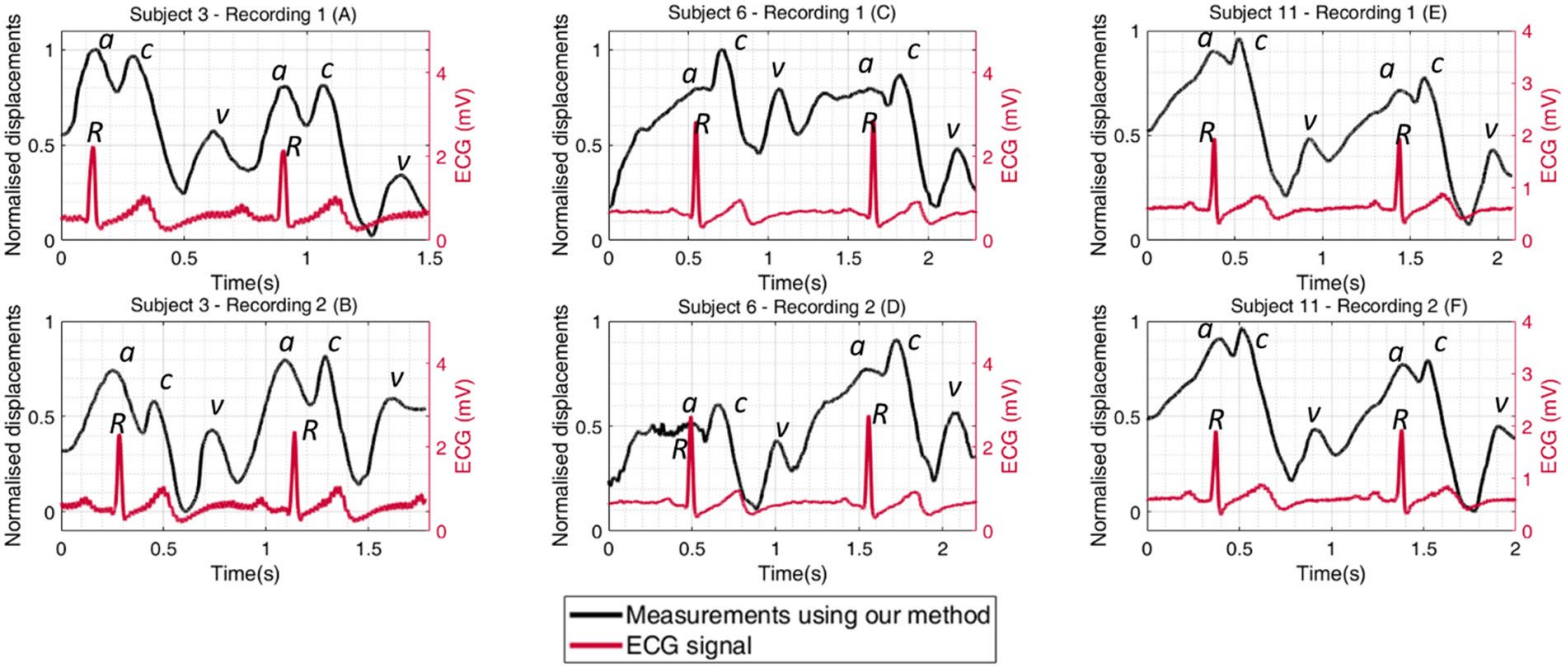
Normalised jugular vein displacement waveforms measured using our method, and synchronised with recorded ECG traces for two recordings in each of three subjects. The R peak of the ECG signal has happened close to the a peak of the JV waveform.

**Figure 12:**
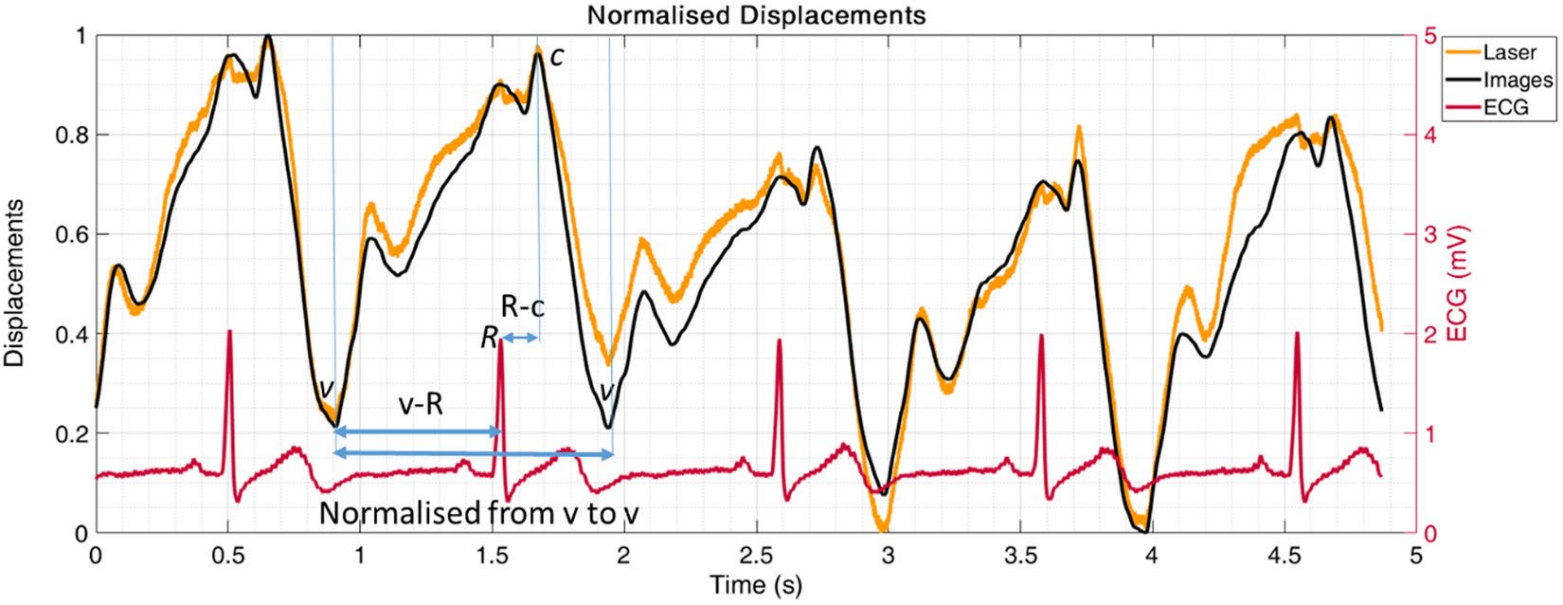
Measured JV waveforms from video recordings for five cardiac cycles of subject 11. The video recordings were synchronised with ECG signals. To perform a comparison between the recordings, two time intervals were defined. The first interval was the time between the v descent of the JV waveforms and the R peak of the ECG signal (v-R), and the second one was the duration between the R peak of the ECG signal and the c peak of the JV waveforms. All the durations were normalized for one cycle duration (v to v).

The measured displacement waveforms and simultaneously recorded ECG traces for three subjects are illustrated in Fig. 11. The displacement waveforms measured using our device show the *R* wave of the ECG traces occurring at a similar time to the *a* peak of JV displacement waveforms (Fig. 11). In order to perform a statistical analysis between the measured waveforms of our subjects, two time intervals were defined in the JV waveforms. The first interval was the time between the *v* descent of the JV waveforms and the *R* peak of the ECG signal (*v-R* in Fig. 12), and the second interval was the duration between the *R* peak of the ECG signal and the *c* peak of the JV waveforms (*R-c* in Fig. 12). It is difficult to perform a direct comparison of the time intervals, since the duration of the cardiac cycle varies within subjects. To address this issue, the *v-R* and *R-c* intervals were normalised using the *v-v* interval of the cardiac cycle (i.e. were divided to the duration from *v* descent in the JV waveform to the next *v* descent as shown in Fig. 12).

Table 2 shows the means and the standard deviations of normalised *v-R* and *R-c* intervals for two recordings from 8 subjects. Each recording consisted of five cycles, from which the *v-R* and *R-c* intervals were manually detected, measured, averaged for one recording, and then normalised using the *v*-*v* interval of the waveform (a sample waveform is shown in Fig. 12). As Table 2 illustrates, the *v-R* intervals were similar for the two recordings from subjects 4, 5, 10, 11, and 16, whereas the *R-c* intervals were similar for the two recordings from subjects 3, 4, 5, 6, 10, 11, and 16. Subject 9 was the only subject who did not have similar *v-R* or *R-c* intervals. The duration of the normalised *v-R* intervals varied from 0.360 in recording 1 of subject 9 to 0.547 in recording 2 of subject 6. The *R-c* intervals ranged from 0.139 in recording 1 of subject 11 to 0.248 in recording 1 of subject 9.

**Table 2:** The average and the standard deviation of the normalised v-R and R-c intervals for 8 subjects who had at least two separate 5 seconds recordings synchronised with the ECG signal (refer to Fig. 12 for the descriptions of the intervals).

The average and the standard deviation of the normalised v-R durations for each subject.			
Subject 3		Subject 4	
Rec. 1	Rec. 2	Rec. 1	Rec. 2
366.5 ± 26.1	418.4 ± 44.3	511.5 ± 16.4	518.2 ± 16.5
**Subject 5**		**Subject 6**	
**Rec. 1**	**Rec. 2**	**Rec. 1**	**Rec. 2**
419.5 ± 32.5	415.7 ± 38.4	476.3 ± 97.6	547.2 ± 20.4
**Subject 9**		**Subject 10**	
**Rec. 1**	**Rec. 2**	**Rec. 1**	**Rec. 2**
360.7 ± 8.0	461.8 ± 15.8	496.3 ± 27.0	469.3 ± 12.5
**Subject 11**		**Subject 16**	
**Rec. 1**	**Rec. 2**	**Rec. 1**	**Rec. 2**
467.6 ± 22.4	443.9 ± 20.5	361.0 ± 7.1	346.5 ± 12.8
**The average and the standard deviation of the normalised R-c durations for each subject**.			
**Subject 3**		**Subject 4**	
**Rec. 1**	**Rec. 2**	**Rec. 1**	**Rec. 2**
205.9 ± 8.7	194.3 ± 12.2	190.7 ± 14.7	211.6 ± 16.1
**Subject 5**		**Subject 6**	
**Rec. 1**	**Rec. 2**	**Rec. 1**	**Rec. 2**
195.7 ± 8.8	203.1 ± 14.7	149.6 ± 37.3	155.7 ± 9.1
**Subject 9**		**Subject 10**	
**Rec. 1**	**Rec. 2**	**Rec. 1**	**Rec. 2**
248.3 ± 10.5	192.1 ± 15.5	196.8 ± 15.8	191.8 ± 9.2
**Subject 11**		**Subject 16**	
**Rec. 1**	**Rec. 2**	**Rec. 1**	**Rec. 2**
139.6 ± 6.1	149 ± 3.7	248.2 ± 11.1	238.8 ± 9.7

## Discussion

We have developed a novel device that can measure, with unprecedented precision, skin displacements caused by the pulsatile flow through the jugular vein. We tested this device on 19 healthy participants and all of the displacement waveforms displayed the expected shape of pressure waveforms reported in the literature (Figs 1 and 9). PPG imaging devices have a demonstrated inability to measure higher order harmonics^12^ and some characteristic features of the JV displacement waveforms^5^ that are important for detection of right heart disease. On the other hand, the method presented here was shown to be able to measure skin deformation waveforms, due to the pulsatile flow of blood through underlying vessels, including all of the characteristic features of the JV displacement waveforms.

Even though a previous study has shown that there is a linear relationship between blood pressure variation and vessel diameter changes^20^, the nonlinear mechanical properties of the soft tissues that lie between the jugular vein and the skin surface prevent a direct conversion of the displacement measurements into realistic estimates of vessel lumen pressure for *in vivo* measurements. For this reason, Casaccia *et al*.^21^ could not directly estimate the intravenous pressure from overlying skin displacements. The difficulty of finding a direct relation between pressure waveforms and displacement waveforms may be due to the lack of understanding of the mechanical properties and thickness of the soft tissues (dermis and adipose) of the skin and vessel, which vary from patient to patient^22^. This knowledge would be required to model how JV pressure waves affect the soft tissues to cause the surface of the skin to move.

In JV pressure waveforms, the *a* peak is reportedly higher than the *c* peak in healthy subjects, yet in our JV displacement waveforms the *c* peak appeared larger than the *a* peak in 18 of the subjects. This is consistent with data gathered by Amelard *et al*., who used a PPG imaging system^5^. The observed differences may be due to the subjects in our studies being positioned in the supine orientation, as opposed to the conventional 45° inclined position (Fig. 1). As the ventricle contracts, the load exerted on the tricuspid valve may be higher when in the supine position. This leads to an elevation in JV pressure^3,23^, and hence a more prominent *c* peak compared to when a subject is positioned at the conventional 45° incline.

The JV pressure waveforms found in the literature usually show the highest point of the *a* wave occurring shortly before *R* peaks of the ECG (Fig. 1), whereas in our displacement waveforms it occurred close to the highest point of the *a* wave (Fig. 11). Note that JV pressure waveforms in most of the studies in the literature were measured with a catheter inserted down the vein into the superior vena cava (very close to the right atrium^24^), while our JV displacement waveforms were measured from an area close to the neck, some distance from the heart, which may explain the observed delay.

The shape of the JV displacement waveforms varies from one heart cycle to the next, as illustrated in Fig. 9. Computing an average over several cycles would help to generate a more consistent waveform. However, simple averaging techniques are inappropriate due to the variable duration of the cardiac cycle. Furthermore, some pathologic features of the waveforms may only be present in some of the cardiac cycles, and they may be masked by averaging. Further studies may be required to find an effective technique for averaging the displacement waveforms over multiple cardiac cycles.

The use of image registration techniques for JV displacement measurements has previously been limited by a lack of accuracy and robustness to noise. The development of the P-SG-GC algorithm has overcome both of these restrictions, enabling estimation of the skin deformations using only intrinsic skin features. However, the accuracy of our method may be degraded by significant noise or saturated pixels. A large region of saturated intensities can interfere with the displacement measurements, as these pixels reduce the algorithm’s ability to compare one image to another, and therefore compute the relative displacements.

The non-contact nature of camera-based methods enables measurements that are not influenced by external forces, a problem when using devices such as ultrasound machines. Our camera-based system also give an advantage of being able to use low cost commodity devices, enabling potential implementation of our method using smartphones or tablets for mobile applications. In addition to its diagnostic use, accurate measurement of the JV pressure waveforms has the benefit of providing useful data about the performance of the heart for cardiac modelling. Computational models of the heart and vessels allow analysis of cardiac mechanics and electrophysiology, as well as more advanced uses such as surgical planning^25^. Such models are ideally informed by clinical data, for both patient-specific modelling and for validation of the models^26^.

## Conclusions

We have developed a novel non-contact imaging method to measure displacements of skin on the neck caused by changes in the JV pressure. To our knowledge, this is the first use of image registration techniques for measurement of JV displacement waveforms. Our method has the potential to be used with common technology that is readily available to clinicians, such as mobile phones or digital cameras. This method can be used to assess cardiac function in the context of a regular clinical examination, and as an aid in the monitoring of patients in remote locations. The next steps are to use our device to measure JV displacement waveforms for patients with known cardiovascular diseases, and to explore relationships between the displacement waveforms measured with our device and the internal pressure of the jugular vein. Ultimately, clinical trials will be required to assess whether this novel technique is suitable for identifying heart disease, and can therefore provide clinically useful diagnostic information.
